# Supplementary material for: Bandgap control in two-dimensional semiconductors via coherent doping of plasmonic hot electrons
Source: Nat Commun. 2021 Jul 15;12:4332. doi: 10.1038/s41467-021-24667-8 (PMC8282635; doi:10.1038/s41467-021-24667-8)
Supplement: Supplementary file 1 — Supplementary Information [file 41467_2021_24667_MOESM1_ESM.pdf]

Bandgap Control in Two-Dimensional Semiconductors via Coherent Doping of Plasmonic Hot Electrons

# - Supplementary Information

Yu-Hui Chen,[1,*] Ronnie R. Tamming,[2,3,4,*] Kai Chen,[2,3,4] Zhepeng Zhang,[5] Fengjiang Liu,[6,7] Yanfeng Zhang,[5] Justin M. Hodgkiss,[2,3,4] Richard J. Blaikie,[2,3,8] Boyang Ding,[2,3,8,†] and Min Qiu[6,7,‡]

[1]*School of Physics, Beijing Institute of Technology, Beijing 10081, China*

[2]*MacDiarmid Institute for Advanced Materials and Nanotechnology*

[3]*Dodd-Walls Centre for Photonic and Quantum Technologies*

[4]*School of Chemical and Physical Sciences, Victoria University of Wellington, Wellington 6012, New Zealand*

[5]*Department of Materials Science and Engineering, College of Engineering, Center for Nanochemistry (CNC), College of Chemistry and Molecular Engineering, Academy for Advanced Interdisciplinary Studies, Peking University, Beijing 100871, China*

[6]*Key Laboratory of 3D Micro/Nano Fabrication and Characterization of Zhejiang Province, School of Engineering, Westlake University, 18 Shilongshan Road, Hangzhou 310024, Zhejiang Province, China*

[7]*Institute of Advanced Technology, Westlake Institute for Advanced Study, 18 Shilongshan Road, Hangzhou 310024, Zhejiang Province, China*

[8]*Department of Physics, University of Otago, PO Box 56, Dunedin 9016, New Zealand*

This PDF file includes:

Section 1. Coupling of Plasmons and Excitons

Section 2. Additional Transient Absorption Spectra of the PC-WS_2 and bare PC

Section 3. Transient Optical Properties of WS_2 monolayers

Section 4. Plasmon-exciton coupling induced charge generation and transfer

Section 5. Transient Analysis of Plasmon-Exciton Polaritons

Section 6. Theoritical Model of Hot Electrons in plasmonic crystal

Section 7. Delayed Photoinduced Absorption with Polaritons

Section 8. Nonlinear responses

Section 1 — Coupling of Plasmons and Excitons

Light-matter coupling builds upon fast energy exchange between photons and excitonic resonances, openning up opportunities for numerous fantastic applications, such as quantum computing and single-photon switch. Here we demonstrate such coupling between plasmon lattice modes in a plasmonic crystal (PC) and WS_2 monolayers (MLs).

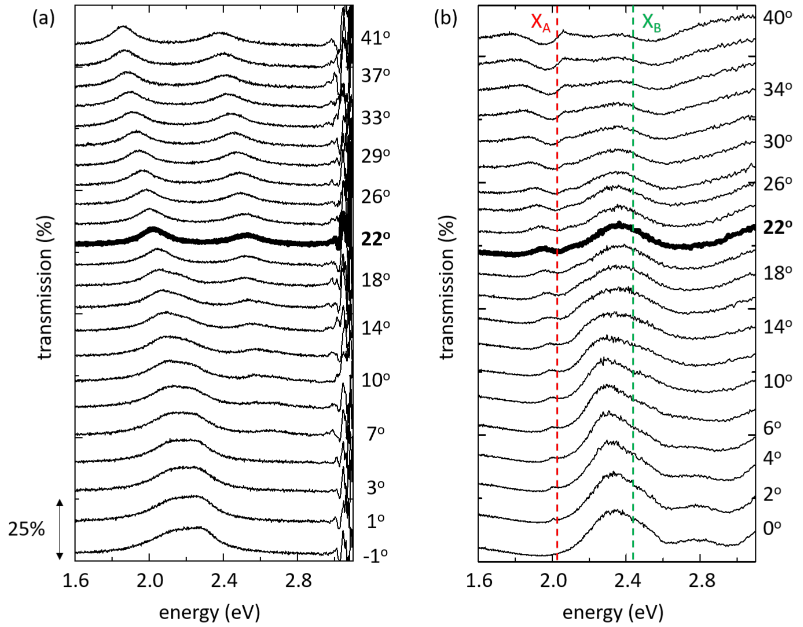

Supplementary Fig. 1. **Angle-resolved tranmission spectra** of (a) a bare PC sample and (b) a PC-WS_2 sample under p-polarized illumination, i.e. the electric field is parallel to the plane of incidence. Dashed lines in panel (b) indicate the spectral positions of exciton A (red) and B (green). The bold curves in both panels indicate the spectrum at $\theta = 22°$.

Supplementary Fig.1 shows the p-polarized angle-resolved transmission spectra of PC samples without and with a WS_2 monolayer on the top. The bare PC spectra [Supplementary Fig.1(a)] show clear dispersions of lattice plasmon resonances, which can be seen from the red-shift of two sets of maxima with increasing incident angles. When integrated with a WS_2 ML [Supplementary Fig.1(b)], the dispersive behaviours of the PC are significantly altered. At the spectral position of exciton A (X_A), the transmission spectra exhibit clear dips, while at the spectral position of exciton B (X_B), the spectra display as maxima, which broaden the spectral features at ~2.4 eV and can be observed from 0° to 40°.

To study the dispersive behaviours of these resonances, we have used the Lorentz oscillator model to fit the lineshapes of the transmission spectra. Supplementary Fig.2(a) shows the trans-

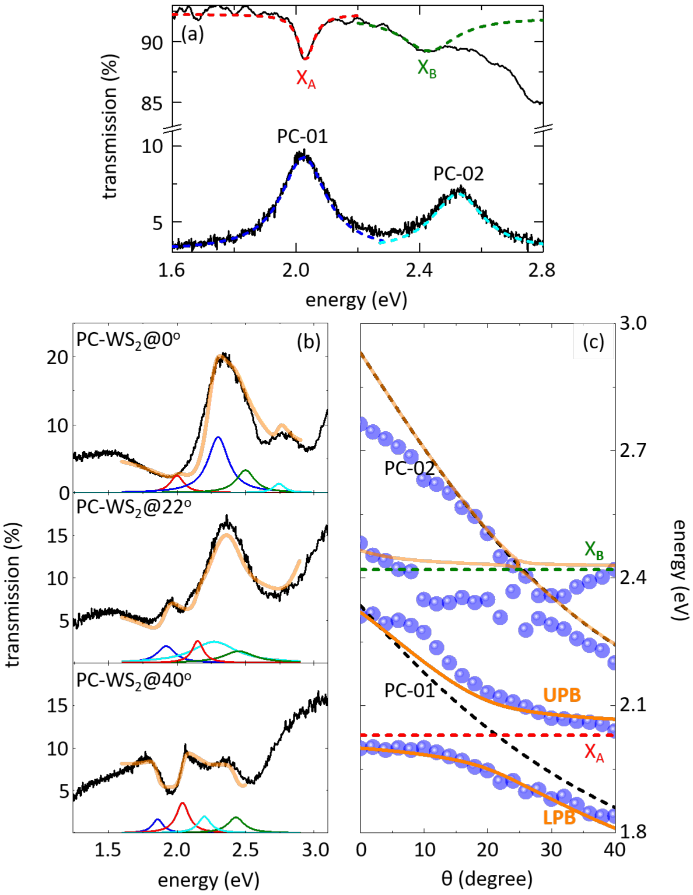

Supplementary Fig. 2. **Analysis of spectral line shape and plasmon-exciton coupling.** (a) the transmission spectrum of a WS_2 monolayer (upper curve) and a bare PC sample (lower curve) measured at $\theta = 22°$ under p-polarized illumination; dotted curves are the fitted results of maxima using Lorentz oscillator models. (b) the p-polarized transmission spectra (black curves) of a PC-WS_2 sample measured at $\theta = 0, 22, 40°$; thick orange curves indicate the fitted results using Eq.S1, and thin curves represent the fitting components, whose amplitudes have been adjusted for visualation. (c) spectral positions of the fitted resonances (solid blue circles) in the PC-WS_2 sample at different illumination angles; the red (green) dashed line indicates the spectral position of exciton A (B); the black dashed curves indicate the simulated dispersions of plasmon resonances, while orange solid curves indicate the fitted dispersions using Eq.S2. Panel (b) and (c) are also presented in Fig. 1 in the main text.

mission spectrum of a WS_2 ML and a bare PC sample at $\theta = 22°$. At this angle, the low-frequency plasmon resonance matches the frequency of X_A, labelled as PC-01 mode; and the high-frequency resonance is labelled as PC-02 mode. Both of them are lattice plasmons propagating along the Air-Ag-sphere interface. (Please see our previous work [1] for more details.) The line shape of these resonances can be fitted using the Lorentz model, with cavity (plasmon) dissipation being $\kappa_1 \approx 180$ meV (PC-01) and $\kappa_2 \approx 200$ meV (PC-02), exciton A (X_A) decay rate being $\gamma_A \approx 60$ meV and X_B decay rate being $\gamma_B \approx 210$ meV.

In order to analyse the coupling between these resonances, we then have fitted the transmission spectra of the PC-WS_2 system [Supplementary Fig.2(b)] using

$$T(\omega) = |t(\omega)|^2 = \left| a + \sum_{\mathrm{j}=1} b_{\mathrm{j}} \frac{\pi \frac{\gamma_{\mathrm{j}}}{2}}{(\omega - \omega_{\mathrm{j}}) - i\frac{\gamma_{\mathrm{j}}}{2}} \right|^2 \tag{S1}$$

where $t(\omega)$ is the transmission coefficient; a and b are constants; ω_{j} and γ_{j} are resonance frequency and linewidth respectively. This fitting model can reproduce spectral lineshapes [$T(\omega)$] resulting from the coupling among multiple Lorentz oscillators. Therefore it can be used to fit the spectra of the PC-WS_2 system, in which 4 sets of resonances (PC-01, PC-02, X_A and X_B) co-exist and interact with each other.

Supplementary Fig.2(b) shows examples of transission spectra at different incident angles. Using Eq.S1 with 4 oscillators, we are able to well reproduce the spectra, including the Fano-like lineshape at $\theta = 40°$. Blue dots in Supplementary Fig.2(c) show the spectral positions of the fitted resonances as a function of incident angles. We note that these resonances exhibit strongly dispersive behaviours, which are then investigated by fitting the dispersions using a (4×4) coupled oscillator model[2]:

$$\begin{pmatrix} \tilde{E}_{\kappa_1}(\theta) & g_{1A} & 0 & g_{1B} \\ g_{1A} & \tilde{E}_{\gamma_A} & g_{2A} & 0 \\ 0 & g_{2A} & \tilde{E}_{\kappa_2}(\theta) & g_{2B} \\ g_{1B} & 0 & g_{2B} & \tilde{E}_{\gamma_B} \end{pmatrix} \begin{pmatrix} \alpha_{\kappa_1}(\theta) \\ \alpha_{\gamma_A}(\theta) \\ \alpha_{\kappa_2}(\theta) \\ \alpha_{\gamma_B}(\theta) \end{pmatrix} = \tilde{E}_p(\theta) \begin{pmatrix} \alpha_{\kappa_1}(\theta) \\ \alpha_{\gamma_A}(\theta) \\ \alpha_{\kappa_2}(\theta) \\ \alpha_{\gamma_B}(\theta) \end{pmatrix} \tag{S2}$$

where $\tilde{E}_\kappa(\theta) = E_{\mathrm{pl}}(\theta) - i\kappa(\theta)$, $\tilde{E}_\gamma = E_X - i\gamma$ and $\tilde{E}_p(\theta)$ denote the complex frequency of plasmon resonances, excitonic resonances and the eigen-frequencies of the coupled system respectively, with the damping factors included; and $|\alpha_\kappa(\theta)|^2$ and $|\alpha_\gamma(\theta)|^2$ refer to the mixing coefficients that describe the fraction of plasmons and excitons in the polariton state. The subscript 1 or 2 and A and B refer to the specific resonators. (Note: To simplify the analysis, the incoherent interaction channel[2] has not been considered in this model.)

Near the intersection point between the dispersive PC-01 mode and the non-dispersive X_A at $\theta = 22°$, we note that the fitted curves [orange curves in Supplementary Fig.2(c)] well match the anti-crossing spectral feature. This is a sign of the formation of plasmon-exciton polaritons, and the two split resonances represent the upper branch (UP) and lower branch (LP) of polaritons. At

θ = 30° (the detuned state), the UP branch approaches to the energy of exciton A, while the LP branch closes to the PC frequency.

In contrast, at the intersection point between PC-02 and X_B (θ = 26°) the anti-crossing behaviour is absent. In addition, the resonance positions near this point slightly drift from the fitting results, which should be induced by the highly lossy nature of the oscillators. In particular, the broad linewidths of PC-02 and X_B may have tempered the accuracy of spectral fitting.

As the result of the dispersion fitting, we then have obtained the strength of coupling between PC-01 mode and X_A being $g_{1A} \approx 87$ meV; the strength of coupling between PC-02 mode and X_B being $g_{2B} \approx 30$ meV; the strength of coupling between PC-01 mode and X_B being $g_{1B} \approx 70$ meV; and the strength of coupling between PC-02 mode and X_A being $g_{2A} \approx 0$ meV.

We first focus on the coupling between PC-01 mode and X_A. Due to the system loss, the coupling strength $g_{1A} \approx 87$ meV yields a ∼140 meV splitting in the PC-WS_2 spectrum at θ = 22°. This fitted result shows $\kappa_1 > 2g_{1A} > \gamma_A$, signifying that the cavity dissipation is faster than both the plasmon-exciton energy exchange rate and the exciton decay. However, the coupling strength can still achieve at $2g_{1A} > (\kappa_1 + \gamma_A)/2$, which, according to the extensively used criteria in studies of plasmon-exciton coupling[2, 3], is within the intermediate coupling regime. In other words, the coherent energy exchange between plasmons and excitons remains effective in the presence of moderate cavity losses, which can be illustrated by analysing the relative degree of mixing between the cavity-plasmon and the exciton states.

In Supplementary Fig.3, we have plotted the mixing coefficients as a function of incident angles, where only shown are the coefficients of LP branch. Supplementary Fig.3(a) shows the coefficients without damping ($\kappa_1 = 0$ and $\gamma_A = 0$). The solid curves start with ∼ 0% and ∼ 100% at θ = 0° for plasmon and exciton respectively and then intersect with each other at $\theta \approx 22°$. The crossing point has a value of 50%, which means that plasmons and excitons make up a 50 − 50 ratio at the tuned state, signifying the establishment of the half-light half-matter hybrid state in the coupled system.

Then we take damping into consideration. As shown in Supplementary Fig.3(a), when plasmons have moderate losses, e.g. $2g_{1A} > (\kappa_1 + \gamma_A)/2$ (where $g_{1A} = 87$ meV, $\kappa_1 = 180$ meV and $\gamma_A = 60$ meV for all angles, mimicking experimental values at the tuned state), the mixing coefficients at the crossing point can still achieve ∼50%. In contrast, when the plasmon damping is increased to $\kappa_1 = 250$ meV with $2g_{1A} < (\kappa_1 + \gamma_A)/2$, the crossing point disappear and the mixing coefficients move apart, indicating a weak coupling state. In other words, the moderate losses in

plasmons do not significantly change the coherent energy exchange nature.

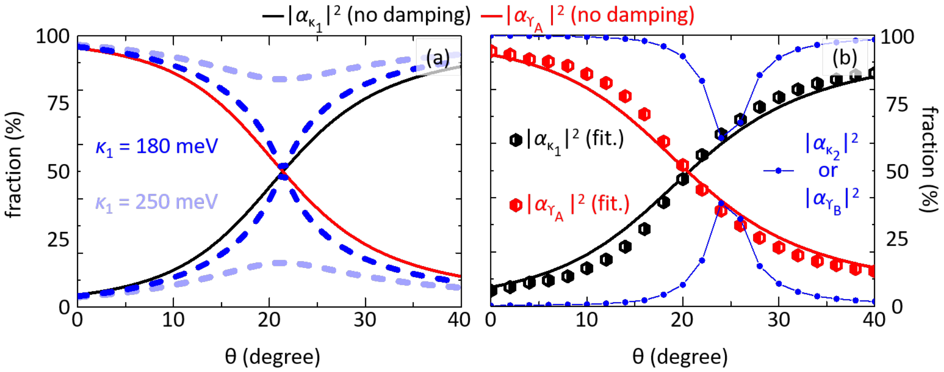

Supplementary Fig. 3. **Mixing coefficients of LPB.** (a) simulated coefficients without (solid curves) and with damping (dahsed curves) that have different magnitudes (γ_A is fixed at 60 meV); (b) the coefficients (solid dots) calculated from the fitted results of measured spectra using Eq.S1 and Eq.S2; black and red dots correspond to the lower branch fitting of PC-01&X_A coupling, while blue dots correspond to the PC-02&X_B coupling. See Supplementary Fig.2(c).

This conclusion can be confirmed by experimentally acquired data. Black and red dots in Supplementary Fig.3(b) demonstrate the coefficients calculated from the fitting parameters of measured spectra, where plasmon damping vary at different angles but hold $\kappa_1 \approx 180$ meV at $\theta \approx 22°$. The coefficients, though slightly drifting from the damping-free curves, can still approach $\sim 50\%$ at the crossing point, suggesting that the coherent energy exchange between PC-01 mode and X_A remains effective in the PC-WS_2 system, which, as will be discussed in Section 6, facilitates hot electron generation.

Now we look into the coupling between PC-02 mode and exciton B. The coupling strength g_{2B} has a low value of ~30 meV, meeting $2g_{2B} \ll \gamma_B < \kappa_2$. This clearly indicates a weak coupling state, which fails to induce a spectral splitting but results in a spectral broadenning at the crossing point between PC-02 mode and X_B. In this case, the mixing coefficients [blue dots in Supplementary Fig.3(b)] also indicate that there is no coherent energy exchange between PC-02 mode and X_B.

What's worth noting is that there is a coupling between PC-01 mode and X_B, although these two resonances are detuned from each other for most incident angles. Specifically, this coupling has a strength of $g_{1B} \approx 70$ meV, only lying in the weak coupling regime. However, this coupling can lead to spectral broadenning and slight shift of the resonant features in PC-WS_2 system at low incident angles ($\theta = 0 - 6°$). In addition, we want to point out that this coupling has negligible influence on the coupling between PC-01 mode and exciton A, since the tuned state of PC-01 and X_A coupling takes place at $\theta = 22°$, far from the angle that enables PC-01's coupling with X_B.

Section 2 - Additional Transient Absorption Spectra of the PC-WS_2 and bare PC

[1] Lineshape analysis of the angle-dependent transient absorption spectra

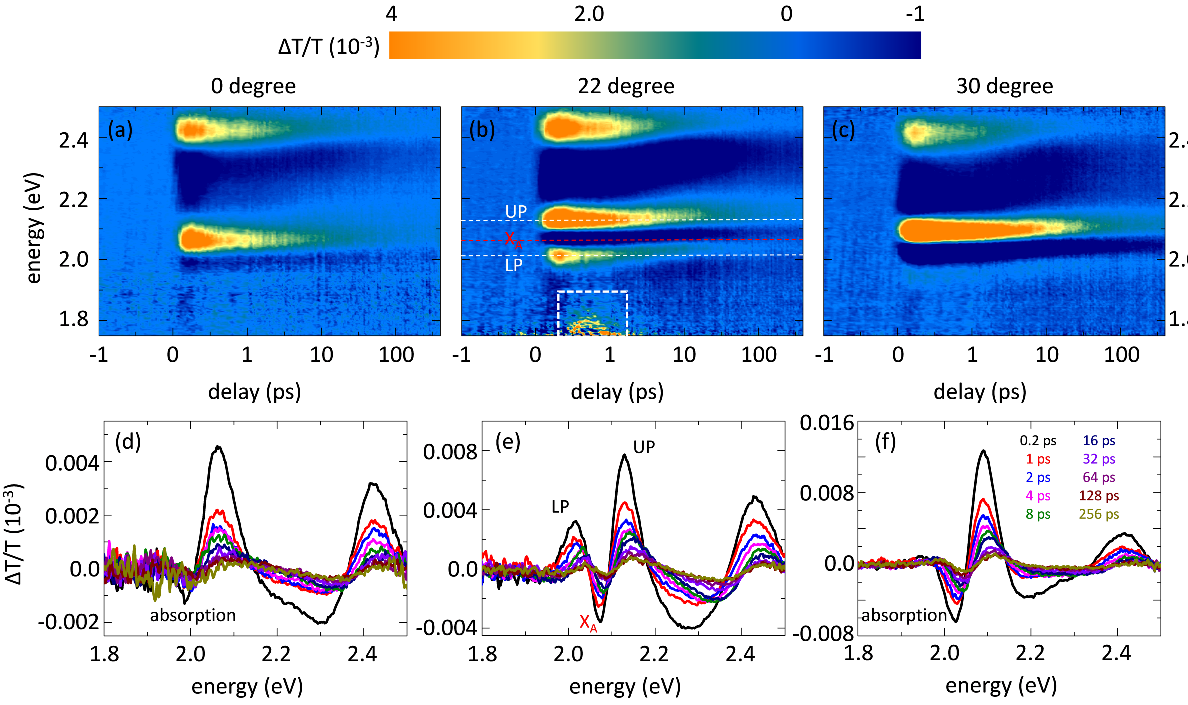

Supplementary Fig. 4. **Transient $\Delta T/T$ spectra of another PC-WS_2 sample** under 17 μJ·cm^{-2} at (a)&(d) $\theta = 0°$, (b)&(e) $\theta = 22°$ and (c)&(f) $\theta = 30°$. Dashed frames in panel b marks the onset of the spectral region of the broad maxima. panel (d), (e) and (f) exhibit the spectra at specific delay time points, which are labelled with different colours as noted in panel (f)

Supplementary Fig.4 shows the additional transient absorption spectra ($\Delta T/T$) taken on a different PC-WS_2 sample from the one discussed in Fig.2 (main text) under 17 μJ·cm^{-2} pump. In general, the tuned state ($\theta = 22°$) and detuned state spectra ($\theta = 0°$ and $\theta = 30°$) all show single-relaxation traces at $\sim$ 2.42 eV, which can be attributed to the bleaching peak of excition B. However, near the spectral position of exciton A ($\sim$ 2.05 eV), these spectra exhibit very different lineshapes and magnitudes. Specifically, the tuned state spectra [Supplementary Fig.4(b)] depict clear double peaks flanking exciton A, while the detuned state spectra [Supplementary Fig.4(a) and 4(c)] all show a single relaxation trace near exciton A but with slightly different peak positions. In particular, the bleaching appears at $\sim$ 2.06 eV for the 0 degree spectra but at $\sim$ 2.09 eV for the 30 degree spectra.

Let us now focus on the splitting at the tuned state [Supplementary Fig.4(b) and Supplementary Fig.4(e)]. The splitting presents as two maxima flanking one minimum. At the delay

time $t = 0.2$ ps, the two maxima appear at 2.02 and 2.13 eV respectively, while the minimum emerges at ~ 2.07 eV. Comparing these values with those in the steady-state measurements [Supplementary Fig.2], we find that the maxima have similar frequencies to those of the UP and LB branches respectively and the minimum roughly corresponds to the excitonic resonance, but all the features exhibit some blue-shifts in the transient spectra. In addition, the splitting (~ 110 meV) in $\Delta T/T$ is smaller than that in the steady-state measurements (~ 140 meV). It is also noted that the smaller splittings together with frequency shifts as compared to steady-state spectra are typically presented at all transient measurements that were taken on different samples [e.g. ~ 95 meV in Fig.2 of the main text and $\sim 90 - 130$ meV with different pump-power in 15].

Furthermore, the bleaching maxima of exciton B in all the tuned and detuned state spectra exhibit no obvious shift , while the maxima/minima associated with exciton A all display pronounced blue-shifts (20 – 30 meV) at late delay times (e.g. > 64 ps). In addition, some spectral positions associated with exciton A exhibit negative magnitudes, e.g. the splitting minimum at ~ 2.07 eV [Supplementary Fig.4(e)], the minimum at 1.99 eV in Fig. 4(d) and the minimum at 2.02 eV in Fig. 4(f). These signify the absorption at this frequency.

We want to point out that the peak shift and negative magnitudes are naturally presented in transient spectra. $\Delta T/T$ are the normalised subtractions between two spectra taken at different delay times. As illustrated in Supplementary Fig.8, resonances may be broadenned and shifted at late delay times due to many-body Auger recombination, i.e. a kind of non-radiative recommbination resulting from exciton-exciton or exciton-electron annihilation. As a result, the feature positions and magnitudes in transient spectra vary from those in steady-state spectra.

What's interesting is that the 22 and 30 degree spectra feature much stronger absorption (negative magnitudes) than do the 0 degree spectra. As explained above, the negative magnitudes are the result of Auger recommbination, which is highly relevant to carrier density. Different absorption magnitudes in spectra of different coupling states suggest that the carrier densities become higher as the coupled system is approaching to the tuned state. [The system at $\theta = 30°$ is closer to the tuned state than the system at $\theta = 0°$, see Supplementary Fig.3.]

Hence we conclude that the coupling between plasmons and excitons results in the different

spectral lineshapes at distinct angles. Approaching to the tuned coupling state enables not only the splittings but also the negative magnitudes and spectral shift in the $\Delta T/T$ transient spectra. What's worth noting is that the tuned state spectra show a broad maximum below 1.85 eV, which presents as a positive gain or negative absorption within 1 ps range. This is identical to result in Fig.2**b** in the main text, indicating the consistency of the PC-WS_2 system and the repeatability of our experiments.

[2] Transient absorption spectra of a bare PC sample at $\theta \approx 22°$

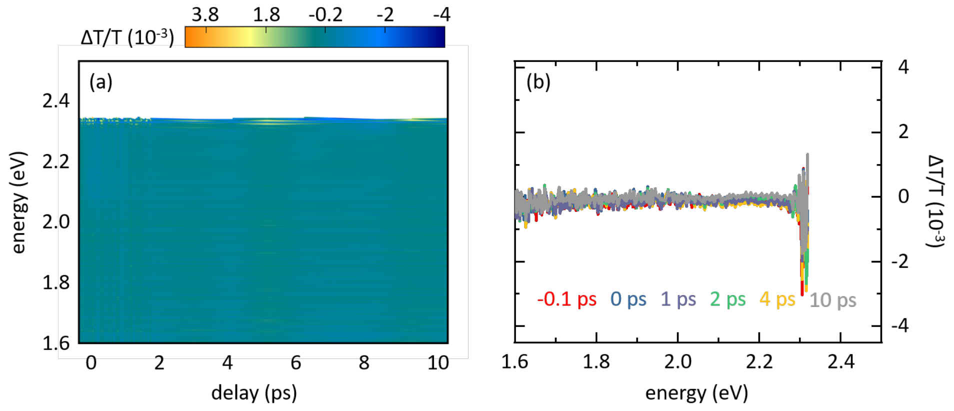

Supplementary Fig. 5. **Transient $\Delta T/T$ spectra of a bare PC** under 20 μJ·cm^{-2} taken at $\theta = 22°$. (a) intensity plot as a function of frequency and delay time; (b) spectra at different delay times.

Supplementary Fig.5 shows the $\Delta T/T$ spectra of a bare PC measured at $\theta = 22°$. As shown in Supplementary Fig.2(c), at this angle, the frequency of plasmon lattice modes is identical to that of the WS_2 ML's exciton A. There are no identifiable spectral features at the frequency ragne of 1.6 – 1.8 eV within 0 – 1 ps, even though the excitation fluence here (20 μJ·cm^{-2}) is a bit higher than the one (12 μJ·cm^{-2}) that enables the broad maxima in PC-WS_2 samples. This result, together with the $\Delta T/T$ spectra of bare WS_2 as shown in Supplementary Fig.6, rule out the possibilities that the broad maxima spectral features at 1.6−1.8 eV are a transient effect of either the plasmonic [4] or the semiconductor system, but indicates that it can only be present when the WS_2 excitons are coupled to the PC.

Section 3 - Transient Optical Properties of WS_2 monolayers

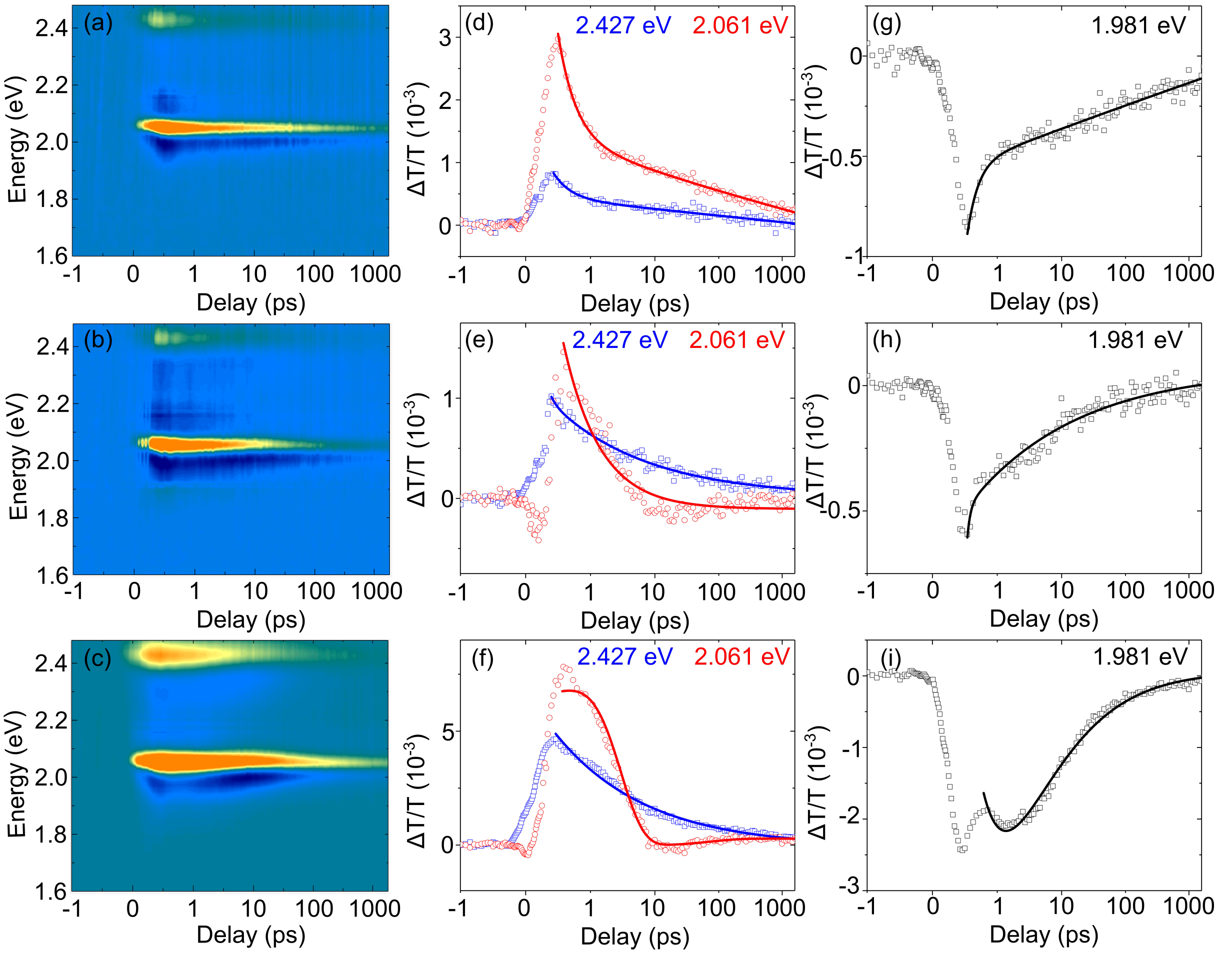

Supplementary Fig. 6. **Transient ΔT/T spectra** of WS_2 MLs under 5 μJ·cm^{-2} (a) (d) and (g), 20 μJ·cm^{-2} (b) (e) and (h), and 100 μJ·cm^{-2} (c) (f) and (i); solid curves are fit results. All measurements were taken at θ = 22°.

In this section we present our reference ultrafast measurements of WS_2 MLs. Supplementary Fig.6 exhibits the ΔT/T spectra of bare WS_2 MLs under different pump intensity. Fitting parameters (red curves in Supplementary Fig.6) associated with exciton A are shown in Table I. For 5μJ·cm^{-2} and 20 μJ·cm^{-2} pump, the photoinduced absorption signal (negative magnitudes manifesting as blue areas) appear almost simulataneoulsy with the bleaching signal (positive magnitudes manifesting as orange area). At high pump fluence (100μJ·cm^{-2}), the absorption maximum (minimum in absolute ΔT/T values) associated with exciton A is postponed as compared to those under 5 and 20 μJ·cm^{-2} pump fluence. The transient cut at E = 1.981 eV (Supplementary Fig.6**i**) directly shows the absorption maximum is delayed to ∼ 3 ps. Such a delay of photoinduced absorption has also been observed in our experiment of tuned polaritons (Fig.2**b** and 2**e** in the main

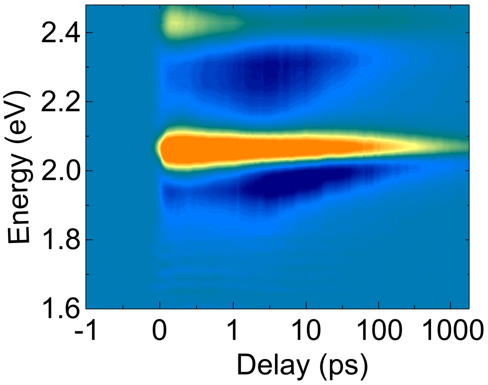

Supplementary Fig. 7. **Transient $\Delta T/T$ spectra** of WS_2 MLs under 69 μJ·cm^{-2} at $\theta = 22°$

text).

Supplementary Table I. List of Fitting Parameters for exciton A in bare WS_2 MLs

components	5μJ·cm^{-2}		20μJ·cm^{-2}		100μJ·cm^{-2}	
τ_1 (ps)	0.51	(73.6%)	0.81	(65.7%)	1.36	(51.8%)
τ_2 (ps)	9.28	(10.7%)	12.65	(21.1%)	8.56	(36.1%)
τ_3 (ps)	213.46	(15.7%)	122.61	(13.3%)	113.97	(12.1%)
τ_{NR} (ps)	1.62		3.68		4.31	

* Return fitting parameters of red solid curves in (Fig. 6**d**, 6e and 6f) using a tri-exponetial function $F(t) = \Sigma_i A_i \mathbf{e}^{-t/\tau_i}$ ($i = 1, 2, 3$);

‡ Percentages, being normalised A_i, refering to population ratios of each fit decay component;

† $\tau_{NR} = \Sigma_\mathrm{i} A_\mathrm{i} \tau_\mathrm{i} / \Sigma_\mathrm{i} A_\mathrm{i}$ with ($i = 1, 2$) represents amplitude-weighed lifetime for polariton formation and non-radiative decays.

According to other experiments with TMDC MLS, the delayed absorption maxima nearby excitons arise when the sample is exposed to high-power pump. In our experiments with bare WS_2 MLs, we can observe such a delayed photoninduced absorption when the pump fluence is larger than 60 μJ·cm^{-2}, as shown Supplementary Fig.7, where pump fluence is 69 μJ·cm^{-2}). Note that, in contrast, for the case of polaritons of our the PC-WS_2 samples (Fig.2**b** in the main text), the delayed absorption maximum appears under only 12 μJ·cm^{-2} pump fluence at the tuned state ($\theta = 22°$).

The delayed photoinduced absorption is widely understood as a result of phonon emission from Auger-type exciton recombinations[5–11]. Particularly, under low pump intensity (upper panel in

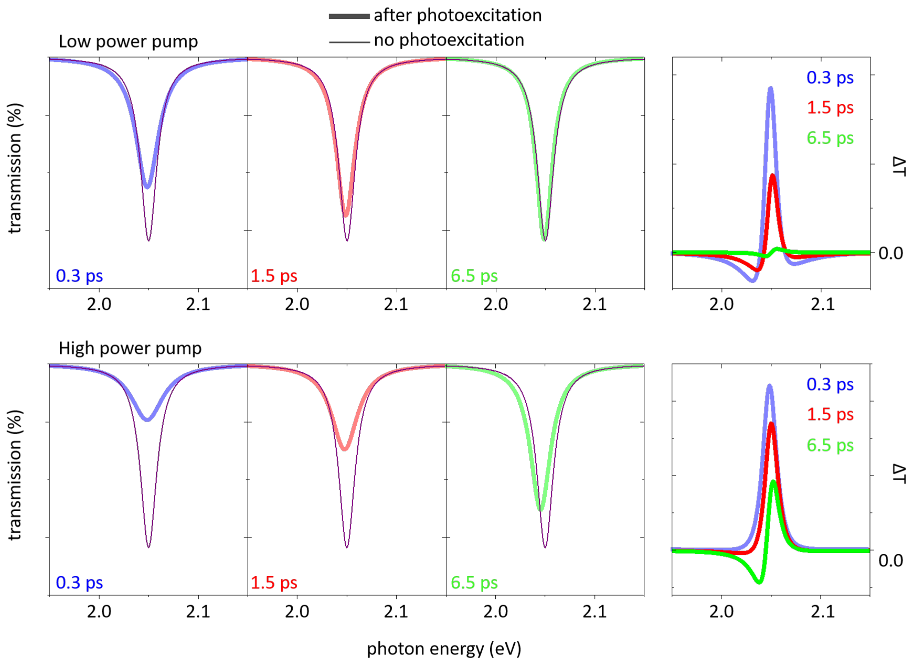

Supplementary Fig. 8. Schematic of the fomation of positive (bleaching) and negative (absorption) values in transmission.

Supplementary Fig.8), during the process of exciton formation (within 1 ps[6]), the photoexcitation can induce Pauli blocking and shift of exciton energy[7], leading to positive and negative values in ΔT at the same time, i.e. the simultaneous coexistence of orange and blue areas near exciton energy in ΔT plots. However, under high-power pump (lower panel in Supplementary Fig.8), the density of excitons is significanly increased, such that many-body effects, e.g. Auger-type exciton-exciton interactions[5], become more significant in exciton relaxations. Such interactions, being effective after 1 ps period of exciton formation[5, 7, 8], produces a large amount of phonons, thermalising semiconductor lattice and inducing Coulomb scattering. These effects lead to spectral broadenning and further shift of exciton energy at a later stage, resulting in the delayed absorption maxima in ΔT spectra. For example, in our experiments with bare WS_2 MLs (Supplementary Fig.6 and 7), the delayed absorption maxima do not appear until pump fluence is larger than 60 μJ·cm^{-2}. This is because high-density excitions induced by strong photoexcitation enhance the probability of exciton-exciton interactions, majority of which decay via Auger-type recombinations, producing a large amount of phonons that thermalise lattice at later times, which broaden and shift absorption lines, featuring blue areas in the ΔT/T spectra.

Section 4 - Plasmon-exciton coupling induced charge transfer and generation

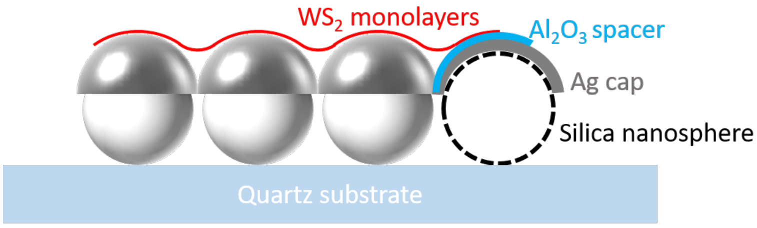

Supplementary Fig. 9. **Schematic of PC-WS_2 samples**, clearly demonstrating the dielectric spacer sandwiched between WS_2 MLs and Ag caps. More detailed morphorlogy of the samples can be found in a previous work[1].

In our study, coherent coupling builds upon fast energy exchange between plasmon waves and 2D excitons, which leads to plasmon-exciton hybridization that is accompanied by charge generation/transfer. As shown in Supplementary Fig.9, the PC-WS_2 hybrid systems acquire a metal-insulator-semiconductor architecture, which is an extensively studied configuration[12–14] that can efficiently harvest hot electrons. In general, two main mechanisms can cause the enhancement of carrier density in the semiconductor lattice.

1. Direct Electron Transfer (DET)

As previously discussed[17, 18], non-radiative decay of plasmon can generate highly energetic hot electrons. As shown in Supplementary Fig.10**a**, depending on their energies, the hot electrons can either tunnel to the WS_2 MLs or overcome the Schottky-like barrier, i.e., being injected into the conduction band of semiconductors. In our structure, there is an ultrathin Al_2O_3 layer (2.5 ± 2 nm) used as a spacer. This insulating layer plays a critical role in forming the energy barrier[16], which can temporarily prevent hot electrons from returning to the plasmonic metals and instead transfer them to the WS_2 lattice. In our experiments, given the large bandgap of Al_2O_3, most of the hot electrons should be transferred to the semiconductors through tunneling the ultrathin spacer[13]. Or in other words, electrons that have energy higher than the tunneling barrier (ϕ_{TB}) can enter the WS_2 lattice.

2. Plasmonic Absorption Enhancement (PAE)

In the PC-WS_2 systems, PAE refers to the process of plasmonically enhanced excitation in the WS_2 lattice. Specifically, as a result of plasmon excitation, local density of optical states of

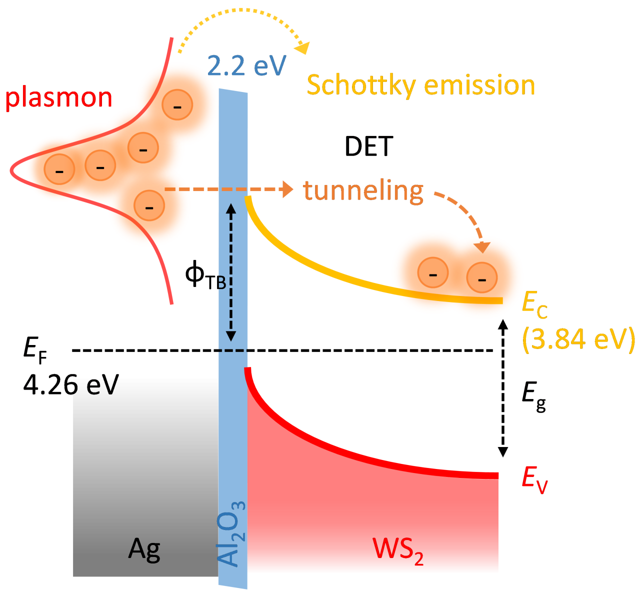

Supplementary Fig. 10. **Schematics of charge transfer/generation** Schematic of DET, direct electron transfer from plasmon waves to WS_2 lattice. E_F refers to the Fermi level, and ϕ_{TB} is the height of tunneling barrier[15]. Numbers refer to band edge energies with reference to vacuum[16]

the systems are elevated due to field enhancement, which then gives rise to the increase of light absorption. If the pump frequency lies in the wide spectral range of plasmons, the pump energy can be more absorbed by the semiconductor, thereby increasing carrier excitation in the semiconductor lattice.

Section 5 - Transient Analysis of Plasmon-Exciton Polaritons

Supplementary Table II. List of Fitting Parameters for PC-WS_2

components	UP @ 22 deg.		LP @ 22 deg.		30 deg.	
τ_1 (ps)	1.08	(48.2%)	1.05	(32.6%)	0.35	(63.0%)
τ_2 (ps)	10.78	(20.7%)	15.67	(37.0%)	5.21	(20.4%)
τ_3 (ps)	83.82	(31.1%)	62.24	(30.4%)	55.38	(16.6%)
τ_{NR} (ps)	3.99		8.83		1.54	

* Parameters in the three colomns corresponding to solid curves of $E = 2.118$ and 2.023 eV (Fig.2**e**) and $E = 2.073$ eV (Fig.2**f**) in the main text respectively;

To better understand the relaxation dynamics, we have used a tri-exponetial function $F(t) = \Sigma_i A_i \mathbf{e}^{-t/\tau_i}$ $(i = 1, 2, 3)$ to fit the decays, similar to the analysis in Table I, shown as solid curves in Fig.2**e** and 2**f** in the main text. The returned fit parameters A_i and τ_i are listed in Table II. Irrespective of energies and detuning states, all relaxations acquire a slow decay constant τ_3 at the scale of tens of picosecond, which is much larger than τ_1 and τ_2. The slower decay arises from radiative electron-hole recombination[9, 11], i.e. photoluminescence (PL) emission, while the sub- to few-picosecond decay in TMDC MLs can be attributed to exciton formation (< 1 ps)[6] together with non-radiative recombination (< 10 ps)[6, 7, 10, 19] induced by Auger-type scattering and defect-assisted relaxations. We note that τ_3 of polaritons in PC-WS_2 samples is significantly smaller than that of bare excitons in WS_2 MLs (Table I), which is consistent with our previous PL experiments[1], where PL from PC-WS_2 is highly enhanced compared to that from WS_2 MLs.

We also use amplitude-weighed parameter $\tau_{NR} = (A_1\tau_1 + A_2\tau_2)/(A_1 + A_2)$ to characterise average lifetime of polariton formation together with non-radiative decays. Specifically at the UP energy, $\sim 70\%$ population decays with a time constant of $\tau_{NR} = 3.99$ ps, which is less than half of the LP time constant (8.83 ps). This result suggests the possibility of rapid transitions from upper to lower branches of polaritons[20], which give rise to phonon emission. Moreover, we also note that τ_{NR} of both UP and LP are much larger than $\tau_{NR} = 1.54$ ps of the detuned polariton at $\theta = 30°$ that is almost identical to τ_{NR} of bare exciton A in WS_2 MLs under low pump intensity (Table I).

As mentioned in the main text, larger τ_{NR} of tuned polaritons compared to the detuned one is a clear indication of hot electron involvement in the plasmon-exciton coupling process[21].

Specifically, after photoexcitation, both plasmons and excitons are excited and alternately transfer energy to each other. Here we introduce this process starting from the exciton side, while the process starting from plasmon excitation undergoes opposite procedures. First, excitons in WS_2 MLs excite plasmons through non-radiative dipole-dipole interaction. Then the excited plasmons transfer energy together with charges (hot electrons) back to the 2D semiconductors. The transferred/generated hot electrons fill unoccupied states in the conduction band of WS_2 MLs. As a result, the absorption of the exciton (polariton) is lower than that without the hot electron injection[22], which gives an bleaching signal in the $\Delta T/T$ curve. On the other hand, the repeated injection/generation of hot electrons makes the $\Delta T/T$ magnitudes decay not as fast as the ones without hot electron injection/generation, further resulting in the extended lifetimes (τ_{NR}) in Fig.2**e** (main text).

Here we briefly discuss the polariton relaxation dynamics in PC-WS_2 systems from the point view of temperature change. It is well known[8] that the spectral positions of excitons shift with the pump-probe time delay, which is induced by the many-body interactions between photo-carriers together with subsequent energy transfer to phonon systems. In this case, not too large lattice temperature changes can be approximated by the resonance frequency shift. Specifically, we can use temperature coefficients of -0.3 meV/K[8] to estimate the the temperature change (ΔTemp) with respect to the energy shift of UP and LP in the PC-WS_2 system.

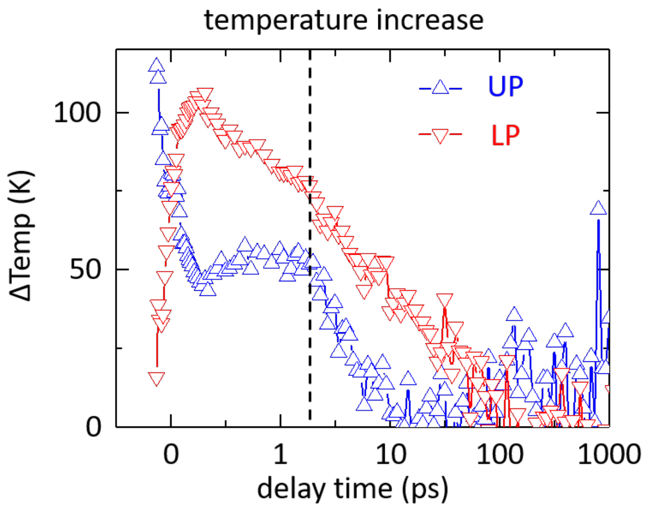

Supplementary Fig. 11. **Lattice temperature analysis** Increase of lattice temperature ΔTemp in the tuned PC-WS_2 system extracted from the energy shift of UP (blue) and LP (red) branches for a pump fluence of 12 μJ·cm^{-2}; The dashed line guides the maximum temperature increase in the lattice.

As shown in Supplementary Fig.11, ΔTemp for UP and LP branches exhibit opposite variation traces within the first 1 ps after photoexcitation, but can all achieve above 100 K. This is because the pump energy (3.1 eV) is much higher than the excitonic resonance (2.05 eV), after photoexcitation, it typically takes 1 ps to form polaritons (excitons) [23]. In the meanwhile, hot electrons repeatedly populate the lattice via plasmon-exciton coupling, temporarily accumulating in the lattice, which is the origin of the broad maxima in the spectral at the $0-1$ ps range, i.e. the giant bandgap renormalisation accompanied by population inversion. At this stage, the system's optical response is dominated by the presence of photoexcited carriers[8] together with hot electrons, and a local temperature model is not that applicable.

We note that there appears a turning point for both UP and LP at the time range of 2 - 3 ps (indicated by the dash line in Supplementary Fig.11). After that, ΔTemp swiftly decreases, suggesting the energy relaxation through both non-radiative and radiative channels. The non-radiative decay of polaritons (excitons) occurs through Auger-type non-radiative recombination at the scales of 10 ps, which can be affected by the injection of hot electrons. The radiative decay takes up to few-hundred picoseconds, leading to photoluminescence (PL) emission. As a result of coupling with plasmon resonances, the PL is enhanced as we have reported in a recent study[1].

Section 6 - Theoritical Model of Hot Electrons in plasmonic crystal

In this section we developed a model to understand the hot-electron generation on the surface of our plasmonic structure. The basic assumptions of the model involves: (a) Electrons in silver are considered as a Fermi gas; (b) Electron-hole excitations, which are created by absorbing energy quanta from plasmonic waves, are considered as a weak perturbation to the Fermi gas. (c) Many body effects that introduce dissipations, such as electron-phonon interaction, are excluded. Because within the 1-ps time scale of the discussed giant bandgap renormalization in our paper, effects from phonons are much less significant.

The dynamics of an electron can be described by

$$\hbar\frac{\partial\rho}{\partial t} = i[\rho, H] - \hbar\Gamma(\rho) \tag{S3}$$

where ρ is the one-particle density matrix, H is the Hamiltonian of an electron, $\Gamma(\rho)$ is the Lindblad loss terms. In matrix notation we have

$$\hbar\frac{\partial\rho_{\mathrm{nm}}}{\partial t} = i\langle n|[\rho, H_0 + V_{\mathrm{opt}}]|m\rangle - \hbar\Gamma_{\mathrm{nm}}\rho_{\mathrm{nm}} \tag{S4}$$

where $\rho_{\mathrm{nm}} = \langle n|\rho|m\rangle$ are the matrix elements of ρ, H_0 is the unperturbed single-particle Hamiltonian, $|n\rangle$ is the eigenstate of H_0 with an eigenenergy ε_{n}, V_{opt} is the interaction of the electron and the driving plasmonic field, and Γ_{nm} is the dephasing rate (When $m = n$, Γ_{nn} is then replaced by the energy relaxation rate). In our model $\Gamma_{\mathrm{nm}} \equiv \gamma_{\mathrm{Drude}}$ is taken from the loss term of Drude model of the Ag dielectric functions.

ρ_{nm} is related to the interaction of moving one electron from a state of energy ε_{m} to a state of energy ε_{n}, and ρ_{nn} stands for the probability of finding the electron in its state $|n\rangle$. When there is no laser input $V_{\mathrm{opt}} = 0$, the electron gas stays in its equilibrium state

$$\begin{aligned} \rho_{\mathrm{nm}}(t) &= 0 \\ \rho_{\mathrm{nn}}(t) &= \rho_{\mathrm{nn}}^{(0)} = f(\varepsilon_{\mathrm{n}})/N = \frac{1}{N}\frac{1}{e^{(\varepsilon_{\mathrm{n}}-\varepsilon_{\mathrm{F}})/k_{\mathrm{B}}T}+1} \end{aligned} \tag{S5}$$

where where $\rho_{\mathrm{nn}}^{(0)}$ is the equilibrium occupation probability, $f(\varepsilon)$ is the Fermi-Dirac distribution, ε_{F} is the Fermi energy level, k_B is the Boltzmann constant and T is the local temperature. Note that

the set of $|n\rangle$ constitutes a complete basis and $\sum_n \rho_{nn} = 1$. The concept of density of state will be introduced to the model later.

For the interaction between electrons and the plasmonic mode,

$$V_{opt} = e\mathbf{r}\mathbf{E}_{loc}e^{-i\omega t} + e\mathbf{r}\mathbf{E}^*_{loc}e^{i\omega t} \tag{S6}$$

where e is the electron charge, $\mathbf{r}$ is the displacement operator, $\mathbf{E}_{loc}$ is local electric field, ω is the frequency of the input light. Under the rotating wave approximation, the matrix elements $\langle n|V_{opt}|m\rangle$ can be written as

$$\langle n|V_{opt}|m\rangle = V_{nm}e^{-i\omega t} + V^*_{nm}e^{i\omega t} = \langle n|e\mathbf{r}\mathbf{E}_{loc}|m\rangle e^{-i\omega t} + \langle n|e\mathbf{r}\mathbf{E}^*_{loc}|m\rangle e^{-i\omega t} \tag{S7}$$

The system is driven away from its equilibrium state by the plasmonic field. We consider the driving as a weak perturbation and the time dependent $\rho_{nm}(t)$ can thus be written as

$$\begin{aligned} \rho_{nm}(t) &= \tilde{\rho_{nm}}(t)e^{-i\omega t}, \quad n > m \\ \rho_{mn}(t) &= \tilde{\rho_{mn}}(t)e^{i\omega t}, \quad n > m \\ \rho_{nn}(t) &= \rho^{(0)}_{nn} + \delta\rho_{nn}(t) + ... \end{aligned} \tag{S8}$$

where $\tilde{\rho_{nm}}(t)$ and $\tilde{\rho_{mn}}(t)$ are slowly-varying functions. By inserting Eq. (S8) into Eq. (S4), we obtain that

$$-i\hbar\omega\tilde{\rho_{nm}} + \dot{\tilde{\rho_{nm}}} = i(\varepsilon_m\tilde{\rho_{nm}} - \varepsilon_n\tilde{\rho_{nm}}) - \hbar\gamma\tilde{\rho_{nm}} + (\tilde{\rho_{nn}} - \tilde{\rho_{mm}})V_{nm} \tag{S9}$$

As the dephasing of electrons in metal are fast enough (within a few femtoseconds), we assume that $\tilde{\rho_{nm}}$ adiabatically follow the incident field, i.e., $\dot{\tilde{\rho_{nm}}} = 0$. In other words $\tilde{\rho_{nm}}$ quickly reaches its steady-state solution

$$\tilde{\rho_{nm}} = \frac{\rho^{(0)}_{mm} - \rho^{(0)}_{nn}}{\hbar\omega - \varepsilon_n + \varepsilon_m + i\hbar\gamma}V_{nm}, \quad n > m \tag{S10}$$

Noting that ρ_{mn} in Eq. (S8) has a time dependence of $e^{i\omega t}$, we then have

$$\tilde{\rho_{mn}} = \frac{\rho^{(0)}_{nn} - \rho^{(0)}_{mm}}{-\hbar\omega - \varepsilon_m + \varepsilon_n + i\hbar\gamma}V_{mn}, \quad n > m \tag{S11}$$

To calculate the population changes $\delta\rho_{\rm nn}$ for those excited electrons , we need to substitute Eq. (S8) into Eq. (S4) and obtain

$$\hbar\frac{\partial}{\partial t}\delta\rho_{\rm nn} = i\langle n|[\rho, V_{\rm opt}]|n\rangle - \hbar\Gamma_n\delta\rho_{\rm nn} \tag{S12}$$

where $\Gamma_{\rm n}$ is the energy relaxation rate of the state $|n\rangle$. By defining $g_{\rm n}$ and $d_{\rm n}$ as the generation rate and the decay rate of the population in $|n\rangle$, we can rewrite the above equation as

$$\frac{\partial}{\partial t}\delta\rho_{\rm nn} = g_{\rm n} - d_{\rm n} \tag{S13}$$

where

$$g_{\rm n} = \frac{i}{\hbar}\langle n|[\rho, V_{\rm opt}]|n\rangle = i\sum_m(\tilde{\rho_{nm}}V_{mn} - V_{nm}\tilde{\rho_{mn}}) \tag{S14}$$

stands for the probability of exciting the electron to a state of energy $\varepsilon_{\rm n}$. As there are in total N electrons in the system, the number-generation rate of electrons to the state $|n\rangle$ is $G_{\rm n} = \sum_{n=1}^{N} g_{\rm n}$. Neglecting those terms oscillating in time with a term of $e^{\pm i\omega t}$ or $e^{\pm 2i\omega t}$ and substituting Eq. (S10) and (S11) into Eq. (S14), we get

$$G_{\rm n} = \sum_{\rm m}[f(\varepsilon_{\rm m}) - f(\varepsilon_{\rm n})][\frac{|V_{\rm nm}|^2\gamma}{(-\hbar\omega - \varepsilon_{\rm n} + \varepsilon_{\rm m})^2 + \hbar^2\gamma^2} + \frac{|V_{\rm nm}|^2\gamma}{(\hbar\omega - \varepsilon_{\rm n} + \varepsilon_{\rm m})^2 + \hbar^2\gamma^2}] \tag{S15}$$

This is the basic equation that we use to calculated the generated hot electrons. For our system $\hbar\omega \approx 2.1$ eV, $E_{\rm F} = 5.76$ eV, $\gamma = 0.02$ eV, and $k_{\rm B}T \ll \hbar\omega$. Here G_n stands for the number of electrons that are generated to the state of energy ε_n per unit time. In order to compute $G_{\rm n}$ we still need to introduce the concept of density of states and deduce the formula of V_{nm}.

Density of States (DOS)

Note that we are interested in the total number of accumulated hot-electrons in the 2D semiconductor. To do that we first need to calculate the generated hot-electron numbers as a function of energy $\delta n(\varepsilon)$, and then integrate $\delta n(\varepsilon)$ over a range $E_{\rm F} + \Delta\phi_{\rm SB} < \varepsilon < E_{\rm F} + \hbar\omega$, where $\Delta\phi_{\rm SB}$ stands for a energy barrier over which electrons can be transferred to the 2D semiconductor. The generated carrier number $\delta n(\varepsilon)$ within a small energy interval $\Delta\varepsilon$ of energy ε is then given by

$$\delta n(\varepsilon)\Delta\varepsilon = \sum_{\rm n} G_{\rm n}\cdot P(\varepsilon_{\rm n}, \varepsilon) \tag{S16}$$

where

$$P(\varepsilon_\mathrm{n}, \varepsilon) = 1, \ \mathrm{for} |\varepsilon_\mathrm{n} - \varepsilon| < \Delta\varepsilon;$$
$$P(\varepsilon_\mathrm{n}, \varepsilon) = 0, \ \mathrm{for} |\varepsilon_\mathrm{n} - \varepsilon| > \Delta\varepsilon. \tag{S17}$$

$P(\varepsilon_\mathrm{n}, \varepsilon)$ works similarly as a delta function and will be used to introduce DOS into our system.

Calculation of $V_{\mathbf{nm}}$

We consider a metallic thin film, as shown in Supplementary Fig.12. The corresponding wave functions of electrons are

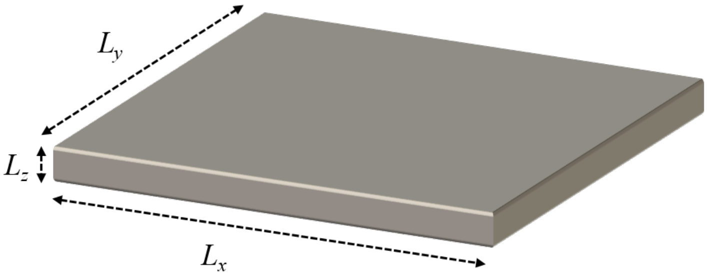

Supplementary Fig. 12. A representation of a metal thin film.

$$\Psi_{n_\mathrm{x}, n_\mathrm{y}, n_\mathrm{z}} = \sqrt{\frac{2}{L_\mathrm{z}}} \frac{1}{L_\mathrm{x} L_\mathrm{y}} \exp(i \frac{n_\mathrm{x}\pi}{L_\mathrm{x}} x) \exp(i \frac{n_\mathrm{y}\pi}{L_\mathrm{y}} y) \sin(\exp i \frac{n_\mathrm{z}\pi}{L_\mathrm{z}} z)$$
$$\varepsilon_\mathrm{n} = \frac{\hbar^2 \pi^2}{2m_0} (\frac{n_\mathrm{x}^2}{L_\mathrm{x}^2} + \frac{n_\mathrm{y}^2}{L_\mathrm{y}^2} + \frac{n_\mathrm{z}^2}{L_\mathrm{z}^2}) \tag{S18}$$

The quantum number n_x, n_y and n_z give a complete description of all quantum states. We then replace n in Eq.(S15) with $\mathbf{n} = (n_\mathrm{x}, n_\mathrm{y}, n_\mathrm{z})$ and rewrite Eq.(S15) as

$$\delta n(\varepsilon)\Delta\varepsilon = \sum_{\mathbf{n}} \sum_{\mathbf{m}} [f(\varepsilon_\mathbf{m}) - f(\varepsilon_\mathbf{n})][\frac{|V_\mathbf{nm}|^2 \gamma}{(-\omega - \varepsilon_\mathbf{n} + \varepsilon_\mathbf{m})^2 + \gamma^2} + \frac{|V_\mathbf{nm}|^2 \gamma}{(\omega - \varepsilon_\mathbf{n} + \varepsilon_\mathbf{m})^2 + \gamma^2} \tag{S19}$$

where $\mathbf{n} = (n_\mathrm{x}, n_\mathrm{y}, n_\mathrm{z})$ lable the quantum numbers in x, y, and z directions. The spectral distribution

of the rate of generated carries can then be written as

$$\begin{aligned}\delta n(\varepsilon)\Delta\varepsilon &= \sum_{\mathbf{n}}\sum_{\mathbf{m}} G_{\mathrm{n}} P(\varepsilon, \varepsilon_{\mathbf{n}}) \\ &= \sum_{\mathbf{n}}\sum_{\mathbf{m}}[f(\varepsilon_{\mathbf{m}}) - f(\varepsilon_{\mathbf{n}})][\frac{|V_{\mathbf{nm}}|^2\gamma}{(-\hbar\omega - \varepsilon_{\mathbf{n}} + \varepsilon_{\mathbf{m}})^2 + \hbar^2\gamma^2} + \frac{|V_{\mathbf{nm}}|^2\gamma}{(\hbar\omega - \varepsilon_{\mathbf{n}} + \varepsilon_{\mathbf{m}})^2 + \hbar^2\gamma^2}]P(\varepsilon, \varepsilon_{\mathbf{n}})\end{aligned} \tag{S20}$$

where $\mathbf{n} = (n_{\mathrm{x}}, n_{\mathrm{y}}, n_{\mathrm{z}})$ lable the quantum numbers of x, y, and z directions.

Substituting Eq. (S18) into Eq. (S7), we can obtain

$$\begin{aligned}\tilde{V_{\mathbf{nm}}} &= \langle \mathbf{n}|ez\mathbf{E}_{\mathrm{z}}|\mathbf{m}\rangle \\ &= \delta_{n_{\mathrm{x}},m_{\mathrm{x}}}\delta_{n_{\mathrm{y}},m_{\mathrm{y}}} eE_z \frac{2L_{\mathrm{z}}}{\pi^2}[\frac{1}{(m_{\mathrm{z}} - n_{\mathrm{z}})^2} - \frac{1}{(m_{\mathrm{z}} + n_{\mathrm{z}})^2}]\end{aligned} \tag{S21}$$

where $\delta_{n_{\mathrm{x}},m_{\mathrm{x}}}$ and $\delta_{n_{\mathrm{y}},m_{\mathrm{y}}}$ are the Kronecker delta function and $\mathbf{E}_{\mathrm{z}}$ is the electric field along z-direction. Equation (S19) can then be reduced as

$$\begin{aligned}\delta n(\varepsilon)\Delta\varepsilon = &\sum_{n_{\mathrm{x}},n_{\mathrm{y}},n_{\mathrm{z}}}\sum_{m_{\mathrm{x}},m_{\mathrm{y}},m_{\mathrm{z}}}[f(\varepsilon_{\mathbf{m}}) - f(\varepsilon_{\mathbf{n}})] \\ &[\frac{|V_{\mathbf{nm}}|^2\gamma}{(-\hbar\omega - \varepsilon_{\mathbf{n}} + \varepsilon_{\mathbf{m}})^2 + \hbar^2\gamma^2} + \frac{|V_{\mathbf{nm}}|^2\gamma}{(\hbar\omega - \varepsilon_{\mathbf{n}} + \varepsilon_{\mathbf{m}})^2 + \hbar^2\gamma^2}]P(\varepsilon, \varepsilon_{\mathbf{n}}) \\ = &\sum_{n_{\mathrm{x}},n_{\mathrm{y}},n_{\mathrm{z}}}\sum_{m_{\mathrm{z}}}[f(\varepsilon_{\mathbf{m}}) - f(\varepsilon_{\mathbf{n}})] \\ &[\frac{\gamma}{(-\hbar\omega - \varepsilon_{\mathbf{n}} + \varepsilon_{\mathbf{m}})^2 + \hbar^2\gamma^2} + \frac{\gamma}{(\hbar\omega - \varepsilon_{\mathbf{n}} + \varepsilon_{\mathbf{m}})^2 + \hbar^2\gamma^2}] \\ &e^2|\mathbf{E}_{\mathrm{z}}|^2\frac{2L_{\mathrm{z}}}{\pi^2}[\frac{1}{(m_{\mathrm{z}} - n_{\mathrm{z}})^2} - \frac{1}{(m_{\mathrm{z}} + n_{\mathrm{z}})^2}]^2 P(\varepsilon, \varepsilon_{\mathbf{n}})\end{aligned} \tag{S22}$$

For our system, we have $E_{\mathrm{F}} \gg \hbar\omega \gg \gamma, k_{\mathrm{B}}T$ ($E_{\mathrm{F}} = 5.76$ eV, $\hbar\omega \approx 2.1$ eV, $\gamma = 0.02$ eV, and at room temperature $k_{\mathrm{B}}T = 0.02$ eV). We can thus make the following simplification

$$\begin{aligned}&\frac{\gamma}{(-\omega - \varepsilon_{\mathbf{n}} + \varepsilon_{\mathbf{m}})^2 + \gamma^2} \rightarrow \pi\delta(-\omega - \varepsilon_{\mathbf{n}} + \varepsilon_{\mathbf{m}}) \\ &\frac{\gamma}{(\omega - \varepsilon_{\mathbf{n}} + \varepsilon_{\mathbf{m}})^2 + \gamma^2} \rightarrow \pi\delta(\omega - \varepsilon_{\mathbf{n}} + \varepsilon_{\mathbf{m}})\end{aligned} \tag{S23}$$

Equation (S22) then becomes

$$\delta n(\varepsilon)\Delta\varepsilon = \sum_{n_x,n_y,n_z}^{|\varepsilon_{\mathbf{n}}-\varepsilon|<\Delta\varepsilon} \sum_{m_z} [f(\varepsilon_{\mathbf{m}}) - f(\varepsilon_{\mathbf{n}})][\pi\delta(-\omega - \varepsilon_{\mathbf{n}} + \varepsilon_{\mathbf{m}}) + \pi\delta(\omega - \varepsilon_{\mathbf{n}} + \varepsilon_{\mathbf{m}})]$$
$$e^2|\mathbf{E}_z|^2 \frac{2L_z}{\pi^2}[\frac{1}{(m_z - n_z)^2} - \frac{1}{(m_z + n_z)^2}]^2 \tag{S24}$$

In the above equation, the $\mathbf{m}$ has $m_x = n_x$ and $m_y = n_y$, and the summation over n_x, n_y, n_z are limited within a range of $|\varepsilon_{\mathbf{n}} - \varepsilon| < \Delta\varepsilon$. For a given set of $\varepsilon_{\mathbf{n}}$ and n_z, there are many possible combinations of n_x and n_y. The number of these combinations are given by the Density of State of a two dimensional electron gas. The DOS of an area A at an energy ε over a range $\Delta\varepsilon$ is

$$\mathrm{DOS}(\varepsilon) = \frac{Am_0}{\pi\hbar^2} \tag{S25}$$

Then the sum over n_x and n_y in Eq. (S24) can be replaced by the DOS

$$\delta n(\varepsilon)\Delta\varepsilon = \frac{Am_0}{\pi\hbar^2}\Delta\varepsilon \sum_{n_z}\sum_{m_z}[f(\varepsilon - \varepsilon_{n_z} + \varepsilon_{m_z}) - f(\varepsilon)]$$
$$[\pi\delta(-\omega - \varepsilon_{n_z} + \varepsilon_{m_z}) + \pi\delta(\omega - \varepsilon_{n_z} + \varepsilon_{m_z})]$$
$$e^2|\mathbf{E}_z|^2 \frac{2L_z}{\pi^2}[\frac{1}{(m_z - n_z)^2} - \frac{1}{(m_z + n_z)^2}]^2 \tag{S26}$$

By replacing

$$\sum_{n}(...) \rightarrow \int dn(...)$$
$$\sum_{m}(...) \rightarrow \int dm(...), \tag{S27}$$

we can rewrite Eq. (S26)

$$\delta n(\varepsilon) = \frac{Am_0}{\pi\hbar^2}\iint dn_z dm_z [f(\varepsilon - \varepsilon_{n_z} + \varepsilon_{m_z}) - f(\varepsilon)]$$
$$[\pi\delta(-\omega - \varepsilon_{n_z} + \varepsilon_{m_z}) + \pi\delta(\omega - \varepsilon_{n_z} + \varepsilon_{m_z}]$$
$$e^2|\mathbf{E}_z|^2 \frac{2L_z}{\pi^2}[\frac{1}{(m_z - n_z)^2} - \frac{1}{(m_z + n_z)^2})]^2 \tag{S28}$$

Note that there are δ functions in Eq.(S28), $\delta n(\varepsilon)$ can then be calculated by performing one-

dimensional integrals. For hot electrons that are defined as electrons with energy $E_F + \Delta\phi_{TB} < \varepsilon < E_F + \hbar\omega$, we obtain the rate of generation of hot electrons by integrating $\delta n(\varepsilon)$ over the energy interval,

$$\int_{E_F+\Delta\phi_{TB}<\varepsilon<E_F+\hbar\omega} \delta n(\varepsilon)d\varepsilon = \frac{1}{\pi^2}\frac{e^2E_F^2}{\hbar}\frac{\hbar\omega - \Delta\phi_{TB}}{(\hbar\omega)^4}|\mathbf{E}_z|^2 A \tag{S29}$$

The rate per area is

$$R_e = \frac{1}{\pi^2}\frac{e^2E_F^2}{\hbar}\frac{\hbar\omega - \Delta\phi_{TB}}{(\hbar\omega)^4}|\mathbf{E}_{norm.}|^2 \tag{S30}$$

where $\mathbf{E}_z$ is replaced by $\mathbf{E}_{norm.}$, which stands for the E field normal to the Al_2O_3-WS_2 interface. These hot electrons are generated from the moment right after the pump pulse, and immediately be transferred to WS_2 layer across the barrier. Such a process lasts for the duration of the pump τ_{pulse} fs. In long time scale, these generated hot electrons are subjected to dissipations, e.g., electron-electron scattering and electron-phonon interaction. However, the observed giant bandgap renormalization in our paper emerges within the first 1 ps, which means that it is a result of the accumulated electrons immediately after the pulse. The number of hot-electrons per area after excitation by a laser-pulse of duration τ_{pulse} is

$$N_e = \frac{1}{\pi^2}\frac{e^2E_F^2}{\hbar}\frac{\hbar\omega - \Delta\phi_{TB}}{(\hbar\omega)^4}|\mathbf{E}_{norm.}|^2\tau_{pulse} \tag{S31}$$

Including losses

Eq.S31 can be used to calculate the hot electron density in PC-WS_2 systems when the pump is on-resonance with plasmon frequency. However, the pump frequency (3.1 eV) in our experiments is higher than the plasmon frequency at the tuned state(2.05 eV), which means that additonal factors in the down-conversion process have to be taken into account in the modelling to obtain an accurate estimation of the hot electron density. We therefore have rewritten Eq.S31 as:

$$N_e = |\mathbf{E_0}|^2 \cdot \mathcal{F} \cdot \frac{1}{\pi^2}\frac{e^2E_F^2}{\hbar}\frac{\hbar\omega - \Delta\phi_{TB}}{(\hbar\omega)^4}\tau_{pulse} \tag{S32}$$

where $\mathbf{E}_0$ is the equivalent incident field at the tuned frequency and $\mathcal{F} = |\mathbf{E}/\mathbf{E}_0|^2$ is the spatial distribution of intensity enhancement at this frequency as depicted in Fig.4**b** in the main text. In the following we will discuss how $\mathbf{E}_{pump}$ (the incident field at the pump frequency) can develop to $\mathbf{E}_0$. In particular, three main factors take effect in this process.

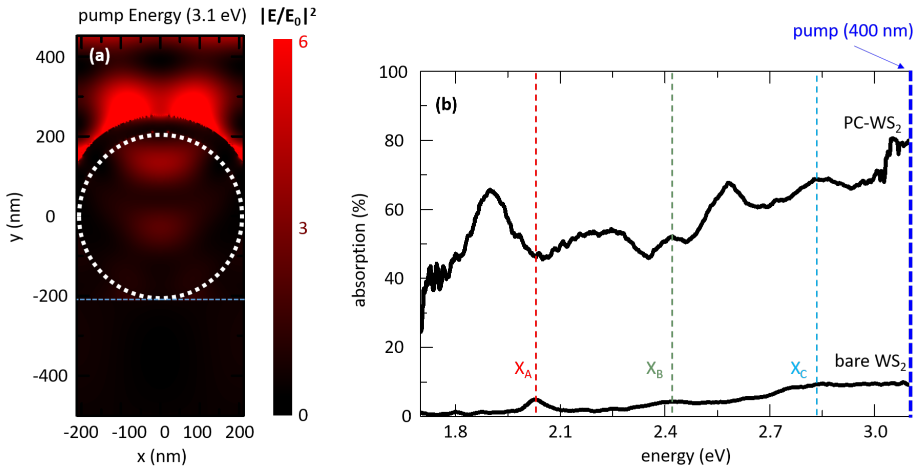

Supplementary Fig. 13. **Light absorption in WS_2 monolayers and PC-WS_2 systems** (a) Field enhancement $|\mathbf{E}/\mathbf{E}_0|$ at the pump energy (3.1 eV or 400 nm). (b) measured absorption spectra of a bare WS_2 ML and a PC-WS_2 sample. The absorption spectra is acquired by $A = 100\% - T - R$, where T refers to transmission and R denotes reflection.

[1] Absorption of pump energy

The pump energy can not be fully absorbed by the system. The optical absorption of a bare WS_2 ML at the pump frequnecy is $\sim$ 10% [Supplementary Fig.13(b)]. When deposited on a plasmonic crystal, the total absorption of the PC-WS_2 system is enhanced to 80%. As shown in Supplementary Fig.13(a), in this system, the intensity enhancement at the pump frequency is no larger than $\sim$7 times at the position of the WS_2 monolayer, which, according to our calculation, results in an average optical absorption of 55% in the monolayer. This is a moderate absorption enhancement compared to that in the bare WS_2 monolayer, but not high enough to induce a Mott-transition as in the work[24].

[2] energy losses in the Down-conversion process

Since the pump photon energy is higher than the exciton frequency, there will be energy dissipation through intraband scattering during the polariton formation, though the total quanta number is still conserved. In particular, the energy that has been transfered to form polaritons follows $\omega_X/\omega_{\text{pump}} \approx 66\%$.

[3] excitation of the plasmonic component in polaritons

In the coupled PC-WS_2 systems, the pump energy is down-converted to form polartions at a lower frequency, but only part of this converted energy will excite the plasmonic com-

ponent of polaritons that generates hot electrons. As revealed by Supplementary Fig.3, the formation of half-plasmon half-exciton hybrid state allows ~ 50% of the converted energy to excite the plasmon part at the tuned state ($\theta = 22°$).

Given that the incident field at the pump frequency can be obtained by:

$$\left|\mathbf{E}_{\text{pump}}\right|^2 = \frac{I_{\text{pump}}}{2\,n\,c\epsilon_0} = \frac{F_{\text{pump}}/\tau_{\text{pulse}}}{2\,n\,c\epsilon_0} \tag{S33}$$

where $F_{\text{pump}} = 12\mu\text{J}\cdot\text{cm}^{-2}$ is the fluence at the pump frequency, $n = 1$ is the refactive index in vacuum, c is the speed of light and ϵ_0 is the vacuum permittivity. The equivalent incident field at the tuned plasmon frequency ($|\mathbf{E}_0|^2$) can be written as:

$$|\mathbf{E}_0|^2 = \frac{\left(F_{\text{pump}} \cdot \eta_{\text{A}} \cdot \eta_{\text{D}} \cdot \eta_{\text{pl}}\right)/\tau_{\text{pulse}}}{2c\epsilon_0} \tag{S34}$$

where $\eta_{\text{A}} = 55\%$ is the absorption coefficient, $\eta_{\text{D}} = 66\%$ is the down-conversion coefficient and $\eta_{\text{pl}} = 50\%$ is the plasmonic coefficient. As a result, Eq.S32 can be rewritten to a new form to estimate the hot electron density that are injected into the WS_2 ML in our experiments:

$$N_{\text{e}} = \frac{\left(F_{\text{pump}} \cdot \eta_{\text{A}} \cdot \eta_{\text{D}} \cdot \eta_{\text{pl}}\right)/\tau_{\text{pulse}}}{2c\epsilon_0} \cdot \mathcal{F} \cdot \frac{1}{\pi^2}\frac{e^2E_{\text{F}}^2}{\hbar}\frac{\hbar\omega - \Delta\phi_{\text{TB}}}{(\hbar\omega)^4}\tau_{\text{pulse}} \tag{S35}$$

which then gives:

$$N_{\text{e}} = \frac{F_{\text{pump}} \cdot \eta_{\text{A}} \cdot \eta_{\text{D}} \cdot \eta_{\text{pl}}}{2c\epsilon_0} \cdot \mathcal{F} \cdot \frac{1}{\pi^2}\frac{e^2E_{\text{F}}^2}{\hbar}\frac{\hbar\omega - \Delta\phi_{\text{TB}}}{(\hbar\omega)^4} \tag{S36}$$

In addition to the factors we have discussed, we take $\phi_{\text{TB}} = 1$ eV for the tunneling barrier at the Ag-Al_2O_3-WS_2 interface. The Al_2O_3 interlayer between the Ag surface and the WS_2 builds an energy barrier at the metal-insulator-semiconductor interface. The height of the barrier naturally depends on the interlayer thickness. As shown in Supplementary Fig.14, the Al_2O_3 layer in PC-WS_2 systems has a nominal thickness of 2.5 ± 2 nm, which, however, is inhomogeneously distributed over the corrugated Ag surface due to vertical evaporation. According to the ref[16],

an Al_2O_3 interlayer with a thickness of 2.1 nm may form a tunneling barrier of $\sim$ 0.8 eV at the metal-WS_2 interface. To cover other losses that are not considered in the calculation, we confine ourselves to a strict criterion of $\phi_{TB} = 1$ eV, which is also the value commonly used in other studies about hot electron transfer[15, 25].

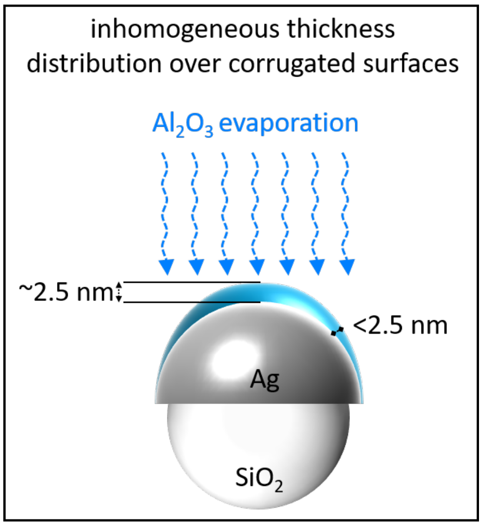

Supplementary Fig. 14. **a schematic of a SiO_2 nanosphere coated with a Ag semi-shell and a thin Al_2O_3 layer.**

Based on Eq.S36, we will be able to calculate the hot electron distribution in WS_2 monolayers in the PC-WS_2 systems, which is plotted in Fig.4**c** as a function of the projected distance along the x-direction in the main text.

Section 7 - Delayed Photoinduced Absorption with Polaritons

Here we discuss the formation of the delayed photoinduced absorption maxima (delayed blue area centred at $\sim$ 1.946 eV in Fig.2**b** of the main text) that are associated with polaritons. Two effects can contribute to the observation:

[1] **Enhanced exciton-electron interaction**

As mentioned in Section 2, the delayed absorption maxima can also be found in bare WS_2 MLs that were pumped under relatively high intensity (e.g. $> 60\mu J{\cdot}cm^{-2}$ in Supplementary Fig.6 and 7), as the result of enhanced exciton-exciton and exciton-electron interactions under high-intensity pump. In the case of tuned polaritons, the probability of exciton-electron interaction is significantly enhanced due to the presence of high-density hot electrons in the lattice[26]. As a result, lots of excitons annihilate through Auger-type recommbination, releasing a large amount of phonons that thermalise the lattice, which can red-shift the exciton energies that leads to the photoinduced absorption at later stages (lower panels in Supplementary Fig.8). This process mainly occurs several ps after the exciton/polariton formation, matching our experimental observations in Supplementary Fig.2**b** and 2**e**.

[2] **Overlap between photoinduced absorption and hot electron population**

As we have discussed in Section 2, with relatively low intensity pump on bare WS_2 MLs, the maxima of photoinduced absorption emerge simultaneously with nearby bleaching maxima (upper panels in Supplementary Fig.8). In the case of tuned polaritons in PC-WS_2 samples, a large number of hot electrons populate the conduction band of the semiconductors, presenting themselves as bleaching signals (positive broad maximum) in $\Delta T/T$ spectra. The frequency range of the population inversion can be extended from the newly formed band edge ($\sim$ 1.6 eV) up to the initial bandgap ($\sim$ 2.15 eV)[24, 27, 28]. With a proper density of hot electrons, this frequency range can overlap with that of the photoinduced absorption, meaning that the absorption minima (negative values) can be compensated by the population inversion (positive values) to a certain degree. Note that in Fig.2**e** (main text), the photoinduced absorption has a turning point at $\sim$ 0.5 ps, which coincides with the accumulation peak of hot electrons (Section 6), verifying this model to some extent.

Section 8 - Nonlinear responses

1. High-power pump induced spectral shift

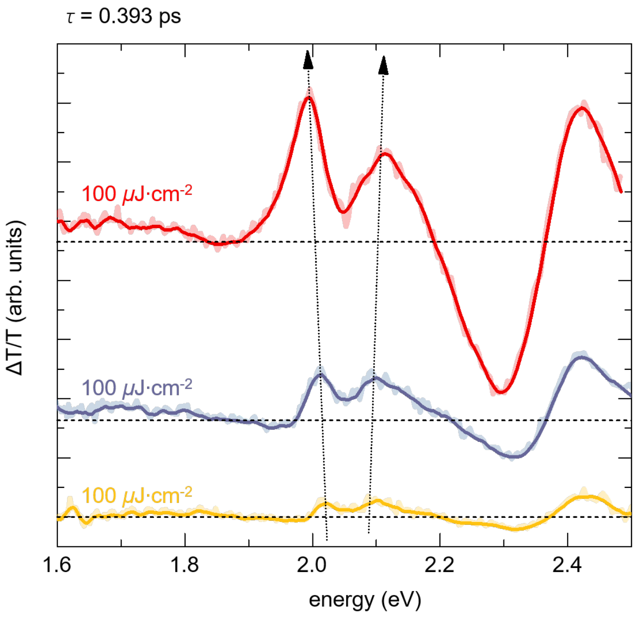

Supplementary Fig. 15. **ΔT/T spectra of the PC-WS$_2$ sample** at θ = 22° under 5 (yellow), 20 (blue) and 100 (red) μJ·cm^{-2} pump fluence. All spectra were cut from ~ 400 fs.

It is noted that as the pump fluence increases (Supplementary Fig.15), the splitting between two maxima becomes larger. This is highly different from a previous work[29] that studied coupling between plasmons and molecular excitons. In their case, the splitting reduces as the pump power increases. Specifically, at a fluence of 44 μJ·cm^{-2}, the splitting is much smaller than in 9 μJ·cm^{-2} case, while completely disappeared at 159 μJ·cm^{-2} fluence. Ref.[29] attributed this finding to saturation of exciton absorption. However in our experiments, excitons are far from the saturation state. Instead we can observe a ~ 35 meV increase of splitting when pump fluence enhances from 5 to 100 μJ·cm^{-2}.

This means that our PC-WS$_2$ systems gain very different nonlinear responses as compared to a recent study on polaritons in WS$_2$ monolayers[30]. We deduce that (i) the ultrasmall mode volumes of plasmonic resonances and (ii) hot electron population in our PC-WS$_2$ systems are responsible for the unusual nonlinearity.

Specifically, the small mode volumes of plasmonic resonances result in large field enhancements, which can certainly enhance the optical nonlinearity of the semiconductor. More importantly, the larger hot electron population under high power pump greatly alters the excitonic prop-

erties of the 2D semiconductor, which can be seen from the relative magnitude change of the split branches. As shown in Supplementary Fig.15, when the pump power is low (5 μJ·cm^{-2}), the lower branch acquires slightly lower magnitude compared to the upper branch, but with increasing pump power, the magnitude of lower branch rapidly rises, reaching up to twice as the magnitude of the upper one under 100 μJ·cm^{-2} pump. This effect is attributed to a red-shift of the exciton frequency under strong pump. Such a shift and the resultant detuning between the frequencies of excitons and plasmons can change the relative weights between the plasmon and the exciton when forming a polariton, and increases the transmission of the lower branch.

2. High-power pump induced delayed maxima

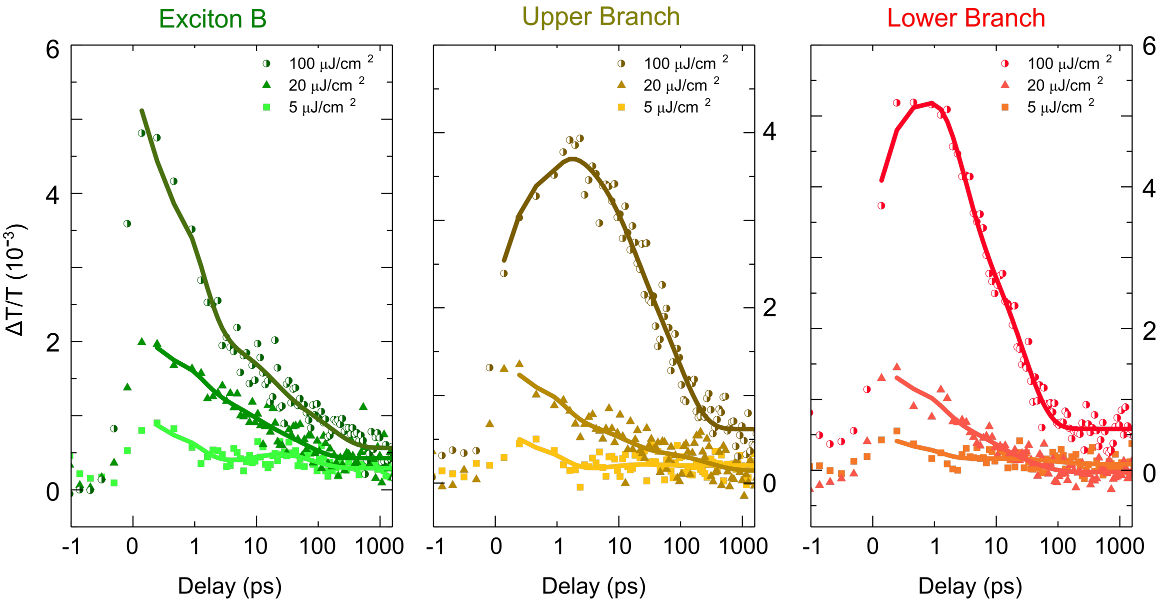

Supplementary Fig. 16. **Pump fluence dependent ΔT/T transient of PC-WS$_2$** at the energy of exciton B (left panel), UB (middle) and LB (right).

We also observe that under high pump fluence (Supplementary Fig.16), UP and LP relaxations exhibit maximum at 3 and 1 ps, respectively, which sharply contrast with the unchanged early maximum ($\sim$ 150 fs) of X_B in the same sample and X_A in bare WS_2 MLs (Supplementary Fig.6), indicating the coupling with plasmon modes gives rise to the delayed maxima, which is highly relevant to hot electron population.

Specifically, as mentioned in Section 3, when plasmons are coupled with excitons, charge transfer/generation accompanies the energy exchange between plasmons and excitons. The period for each energy/charge exchange cycle is very short ($\sim$ 30 fs), which is at the same scale of the injection time of hot electrons (from plasmonic structures to 2D semiconductors) in a recent study[22].

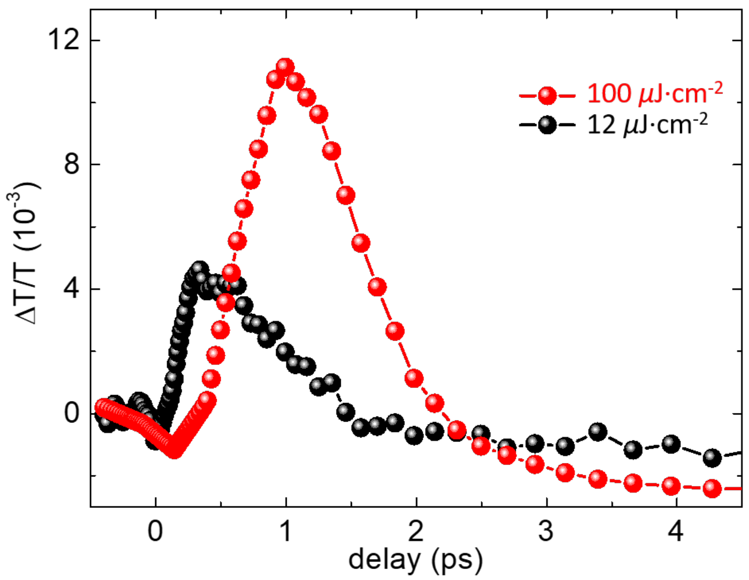

Supplementary Fig. 17. **Pump fluence dependent $\Delta T/T$ transients** spectrally integrated (1.65 – 1.75 eV) at the frequency range of the broad maximum of the PC-WS_2 sample. Please note that there is a ±100 fs error for the starting point of zero probe delay, given that these data were collected from different measurements.

It means that in the intermediate coupling regime, hot electrons frequently populate in the lattice. In the meanwhile, the Al_2O_3 insulator prevents electrons from returning back to the metals, leading to the temporary accumulation of electrons in the lattice. The hot electron population at early periodic cycles (within 100 fs) of energy exchange is significantly intensive as compared to later cycles[31], which result in the short presence time of the broad maxima (1 - 2 ps) in Fig.2 and Fig.3 in the main text.

We also note that under 100 μJ·cm^{-2} pump, UP and LP relaxations manifest maxima at different time points, while this difference is absent under low power pump (Supplementary Fig.16). This results from the faster relaxation of LP than that of UP, especially under the high pump power. For example, τ_2 and τ_3 for UP polartions are 13.32 ps and 95.96 ps respectively, while the LP relaxation shows much shorter lifetime $\tau_2 = 2.40$ ps and $\tau_3 = 27.27$ ps. This phenomenon has been discovered and discussed in our[1, 32] and others'[20] recent studies, where the photoluminescence (PL) intensity of only the LP branch shows nonlinear enhancement with increasing pump power but not the UP branch. Upon high power illumination, hot electrons populates the semiconductor lattice, which competes with both UP and LP relaxation. As a result, the LP branch, which has higher decay rates, shows maximum at a earlier time than does the UP branch.

As shown in Supplementary Fig.17, the peaking time of broad maximum is also dependent

on pump fluence. For example, for 100μJ·cm^{-2} pump (red curve), the maximum peaks at 0.96 ps; while for 12μJ·cm^{-2} pump (black curve), the maximum peaks at 0.33 ps; and they all rapidly decay after the maxima. Because the accumulation of hot electrons naturally competes with the relaxation dynamics of excitons, the peaks with hot electron injections typically appear later than the relaxation maxima of bare excitons (Supplementary Fig.6**c** and 6**f**) typically appear earlier than the hot electron peaks. Specifically, in the case of low-power pump, the electron density is relatively low (Supplementary Fig.17), only capable of slightly affecting the exciton/polariton relaxation, i.e. extended lifetimes of polaritons (τ_{NR}, Section 4). In contrast, for high-power pump, the electron density are highly enhanced, which can significantly slow down the formation of relaxation peaks, or namely delaying the maxima of polariton peaks (Supplementary Fig.16).

REFERENCES

* These authors contributed equally

† boyang.ding@otago.ac.nz

‡ qiu_lab@westlake.edu.cn

[1] B. Ding, Z. Zhang, Y.-H. Chen, Y. Zhang, R. J. Blaikie, and M. Qiu, ACS Nano **13**, 1333 (2019).

[2] W. Wang, P. Vasa, R. Pomraenke, R. Vogelgesang, A. De Sio, E. Sommer, M. Maiuri, C. Manzoni, G. Cerullo, and C. Lienau, ACS Nano **8**, 1056 (2014).

[3] G. Khitrova, H. M. Gibbs, M. Kira, S. W. Koch, and A. Scherer, Nat. Phys. **2**, 81 (2006).

[4] B. Ding, C. Hrelescu, N. Arnold, G. Isic, and T. A. Klar, Nano Lett. **13**, 378 (2013).

[5] T. Jiang, R. Chen, X. Zheng, Z. Xu, and Y. Tang, Opt. Express **26**, 859 (2018).

[6] F. Ceballos and H. Zhao, Adv. Funct. Mater. **27**, 1604509 (2016).

[7] E. J. Sie, A. Steinhoff, C. Gies, C. H. Lui, Q. Ma, M. R?sner, G. Sch?nhoff, F. Jahnke, T. O. Wehling, Y.-H. Lee, J. Kong, P. Jarillo-Herrero, and N. Gedik, Nano Lett. **17**, 4210 (2017).

[8] C. Ruppert, A. Chernikov, H. M. Hill, A. F. Rigosi, and T. F. Heinz, Nano Lett. **17**, 644 (2017).

[9] H. Wang, C. Zhang, and F. Rana, Nano Lett. **15**, 339 (2015), arXiv:1409.4518.

[10] P. D. Cunningham, A. T. Hanbicki, K. M. McCreary, and B. T. Jonker, ACS Nano **11**, 12601 (2017).

[11] M. Amani, D.-H. Lien, D. Kiriya, R. Addou, A. Azcatl, J. Noh, S. R. Madhvapathy, R. Addou, S. Kc, M. Dubey, K. Cho, R. M. Wallace, S.-c. Lee, J.-h. He, J. W. A. Iii, X. Zhang, E. Yablonovitch, and A. Javey, Science (80-.). **350**, 1065 (2015).

[12] S. K. Cushing, J. Li, F. Meng, T. R. Senty, S. Suri, M. Zhi, M. Li, A. D. Bristow, and N. Wu, J. Am. Chem. Soc. **134**, 15033 (2012).

[13] F. P. García De Arquer, A. Mihi, D. Kufer, and G. Konstantatos, ACS Nano **7**, 3581 (2013).

[14] A. Furube and S. Hashimoto, NPG Asia Mater. **9**, e454 (2017).

[15] X. T. Kong, Z. Wang, and A. O. Govorov, Adv. Opt. Mater. **5**, 1 (2017).

[16] S. Zheng, H. Lu, H. Liu, D. Liu, and J. Robertson, Nanoscale **11**, 4811 (2019).

[17] C. Clavero, Nat. Photonics **8**, 95 (2014).

[18] M. L. Brongersma, N. J. Halas, and P. Nordlander, Nat. Nanotechnol. **10**, 25 (2015).

[19] G. Aivazian, H. Yu, S. Wu, J. Yan, D. G. Mandrus, D. Cobden, W. Yao, and X. Xu, 2D Mater. **4**,

025024 (2017).

[20] M. E. Kleemann, R. Chikkaraddy, E. M. Alexeev, D. Kos, C. Carnegie, W. Deacon, A. C. De Pury, C. Große, B. De Nijs, J. Mertens, A. I. Tartakovskii, and J. J. Baumberg, Nat. Commun. **8**, 1296 (2018), arXiv:1704.02756.

[21] A. Boulesbaa, V. E. Babicheva, K. Wang, I. I. Kravchenko, M. W. Lin, M. Mahjouri-Samani, C. B. Jacobs, A. A. Puretzky, K. Xiao, I. Ivanov, C. M. Rouleau, and D. B. Geohegan, ACS Photonics **3**, 2389 (2016).

[22] H. Shan, Y. Yu, X. Wang, Y. Luo, S. Zu, B. Du, T. Han, B. Li, Y. Li, J. Wu, F. Lin, K. Shi, B. K. Tay, Z. Liu, X. Zhu, and Z. Fang, Light Sci. Appl. **8** (2019), 10.1038/s41377-019-0121-6.

[23] F. Ceballos, Q. N. Cui, M. Z. Bellus, and H. Zhao, Nanoscale **8**, 11681 (2016), arXiv:1607.04856.

[24] A. Chernikov, C. Ruppert, H. M. Hill, A. F. Rigosi, and T. F. Heinz, Nat. Photonics **9**, 466 (2015).

[25] L. V. Besteiro, X. T. Kong, Z. Wang, G. Hartland, and A. O. Govorov, ACS Photonics **4**, 2759 (2017).

[26] V. Shahnazaryan, I. Iorsh, I. A. Shelykh, and O. Kyriienko, Phys. Rev. B **96**, 115409 (2017).

[27] L. Meckbach, T. Stroucken, and S. W. Koch, Appl. Phys. Lett. **112** (2018), 10.1063/1.5017069.

[28] S. Park, N. Mutz, T. Schultz, S. Blumstengel, A. Han, A. Aljarb, L. J. Li, E. J. List-Kratochvil, P. Amsalem, and N. Koch, 2D Mater. **5** (2018), 10.1088/2053-1583/aaa4ca.

[29] P. Vasa, R. Pomraenke, G. Cirmi, and E. D. Re, ACS Nano **4**, 7559 (2010).

[30] F. Barachati, A. Fieramosca, S. Hafezian, J. Gu, B. Chakraborty, D. Ballarini, L. Martinu, V. Menon, D. Sanvitto, and S. Kéna-Cohen, Nat. Nanotechnol. **13**, 906 (2018), arXiv:1803.04352.

[31] P. Vasa, W. Wang, R. Pomraenke, M. Lammers, M. Maiuri, C. Manzoni, G. Cerullo, and C. Lienau, Nat. Photon. **7**, 128 (2013).

[32] J. Qin, Z. Zhang, Y.-H. Chen, Y. Zhang, R. Blaikie, B. Ding, and M. Qiu, Phys. Rev. Lett. **124**, 63902 (2020), arXiv:1811.01598.
